# Quantitative Super-Resolution Imaging for the Analysis of GPCR Oligomerization

**DOI:** 10.3390/biom11101503

**Published:** 2021-10-12

**Authors:** Megan D. Joseph, Elena Tomas Bort, Richard P. Grose, Peter J. McCormick, Sabrina Simoncelli

**Affiliations:** 1 London Centre for Nanotechnology, University College London, London WC1H 0AH, UK; megan.joseph.20@ucl.ac.uk; 2 Centre for Tumour Biology, Barts Cancer Institute, Queen Mary University of London, London EC1M 6BQ, UK; e.tomasbort@qmul.ac.uk (E.T.B.); r.p.grose@qmul.ac.uk (R.P.G.); 3 Centre for Endocrinology, William Harvey Research Institute, Barts and The London School of Medicine and Dentistry, Queen Mary University of London, London EC1M 6BQ, UK; p.mccormick@qmul.ac.uk; 4 Department of Chemistry, University College London, London WC1H 0AJ, UK

**Keywords:** super-resolution, DNA-PAINT microscopy, qPAINT, G-protein coupled receptors, purinergic receptor Y2 (P2Y_2_), oligomerization

## Abstract

G-protein coupled receptors (GPCRs) are known to form homo- and hetero- oligomers which are considered critical to modulate their function. However, studying the existence and functional implication of these complexes is not straightforward as controversial results are obtained depending on the method of analysis employed. Here, we use a quantitative single molecule super-resolution imaging technique named qPAINT to quantify complex formation within an example GPCR. qPAINT, based upon DNA-PAINT, takes advantage of the binding kinetics between fluorescently labelled DNA imager strands to complementary DNA docking strands coupled to protein targeting antibodies to quantify the protein copy number in nanoscale dimensions. We demonstrate qPAINT analysis via a novel pipeline to study the oligomerization of the purinergic receptor Y2 (P2Y_2_), a rhodopsin-like GPCR, highly expressed in the pancreatic cancer cell line AsPC-1, under control, agonistic and antagonistic conditions. Results reveal that whilst the density of P2Y_2_ receptors remained unchanged, antagonistic conditions displayed reduced percentage of oligomers, and smaller numbers of receptors in complexes. Yet, the oligomeric state of the receptors was not affected by agonist treatment, in line with previous reports. Understanding P2Y_2_ oligomerization under agonistic and antagonistic conditions will contribute to unravelling P2Y_2_ mechanistic action and therapeutic targeting.

## 1. Introduction

G-protein coupled receptors (GPCRs) are the largest family of cell surface receptors in eukaryotic cells. These seven-transmembrane receptors have influence in physiological events such as cell to cell communication, immune responses, nerve transmission and even hunger and sleep regulation [1,2,3]. The role of GPCRs in diseases such as rheumatoid arthritis, heart disease, cancer, obesity, and neurodegenerative disorders accentuates the need to investigate this family of receptors further. More than a third of all drugs approved by the FDA target GPCRs [4] but often such drugs have a variety of poorly understood mechanisms, as a recent example surrounding opioid receptor agonists illustrates [5,6,7,8]. Understanding these mechanisms, and their influence on GPCR activation, is paramount to improving GPCR drug development.

GPCRs have been shown to form dimers and/or oligomers, where the receptor is present in groups of two or more receptors of the same or different kind. There are several reports of oligomers affecting ligand-binding pharmacology, function, trafficking, and internalisation compared to their monomeric form [9,10]. Several experimental techniques, ranging from traditional biochemical approaches to biophysical ensemble methods based on resonance energy transfer (RET), such as fluorescence resonance energy transfer (FRET) and bioluminescence resonance energy transfer (BRET), have been key to observing the formation of dimers and oligomers [11,12]. However, one of the main issues with these techniques is that they cannot provide information on the size of the oligomeric complexes, nor on the location of the GPCRs in the cell. Over the years, different optical microscopy approaches, based on molecular brightness analysis, have been proposed to tackle some of these shortcomings [13,14,15]. However, they provide average oligomerization information, and it is not possible to extract the precise stoichiometry of specific molecular complexes. A suitable technique to provide information on the oligomeric state of GPCRs and their dynamics down to the single-molecule level is single-molecule tracking [16,17,18]. Yet, this typically requires receptor densities that are orders of magnitude lower than those occurring in natural settings. The relevance of this shortcoming is particularly important when investigating GPCR oligomerization in certain cancer cell lines, where the receptors are overexpressed.

**Scheme 1:**
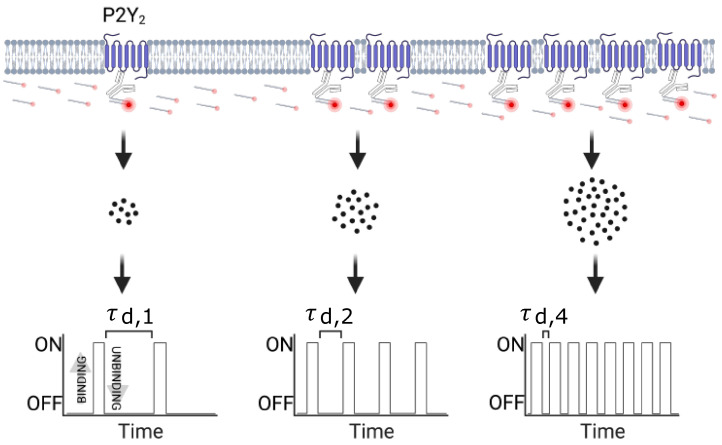
Overview of qPAINT analysis pipeline (created with BioRender.com, BioRender, Toronto YTO, Canada).

Here, we present a novel single-molecule based approach to study the oligomerization of a rhodopsin-like GPCR, the purinergic receptor Y2 (P2Y_2_), in a pancreatic cancer cell line with high expression of the receptor [19]. Our approach is based on the most recently developed single-molecule localization microscopy (SMLM) method named DNA-PAINT [20] (a variation of point accumulation for imaging in nanoscale topography) in combination with an imaging analysis pipeline suitable for protein quantification. DNA-PAINT is an SMLM technique that relies on the repetitive binding between two short complementary single-stranded DNAs, one conjugated to a fluorescent dye (imager strand) and the other chemically coupled to either a primary or secondary antibody targeting the protein of interest (docking strand). These short-lived transient events (millisecond to second range) create the necessary blinking required for SMLM, allowing the localization of the position of single molecules with nanometer precision. Over the course of the experiment, these cumulative DNA binding events form a cluster of single molecule localizations within the true position of the biological target, as illustrated in Scheme 1. Depending on the DNA pair’s binding kinetics in particular the association rate, *k*_on_, and the imager DNA strand concentration, *c_i_*, each docking strand is visited by an imager at a frequency given by (*k*_on_ *c_i_), which corresponds to the inverse of the dark time for a single docking strand (i.e., length of time that a docking strand is not bound to an imager strand between binding events) [21]. The frequency of the imagers binding to their docking strand scales linearly with the number of docking strands, and this is the principle of the quantitative analysis known as qPAINT (Scheme 1). The predictable binding kinetics between imager and docking strands make qPAINT suitable to accurately correlate the frequency of single-molecule events with the underlying number of labelled molecular targets [21], overcoming ‘overcounting’ artifacts observed with other SMLM techniques.

To demonstrate the potential of qPAINT to study GPCRs oligomerization status, we investigated the nanoscale distribution of the P2Y_2_ receptor, a member of the d subgroup of the family A of GPCRs, in the cancer cell line AsPC-1, as it endogenously expresses high levels of this receptor and single cells are easily imaged due to its low levels of cell grouping [19]. P2Y_2_ has been related to immune regulation, bone mineralisation, intraocular pressure, HIV-1 infection, and cancer metastasis and proliferation [22,23,24,25]. P2Y_2_ has recently gained traction due to its role in several cancers such as breast, head and neck, prostate, and pancreatic [19,25,26,27]. This receptor, which can interact with adenosine triphosphate (ATP) and uridine triphosphate (UTP), is known to homo-dimerise and homo-oligomerise [28,29]. When applied to P2Y_2_, qPAINT revealed the molecular densities and nano- and micro-scale spatial arrangements of GPCRs down to the single molecule level, as well as their modulation in response to agonist and antagonist treatment. Understanding the natural oligomerisation state of P2Y_2_ and the effect of agonists and antagonists could inform the mechanist action of P2Y_2_ and aid in targeting it therapeutically.

## 2. Materials and Methods

### 2.1. Cell Culture

AsPC-1 cells, a cell line derived from pancreatic ductal adenocarcinoma [30] and PS-1 cells, which are immortalised human pancreatic stellate cells [31] were a kind gift from Prof. Hemant Kocher (Barts Cancer Institute, London, UK). Cells were cultured using RPMI-1640 medium (Gibco, Amarillo, TX, USA) for AsPC-1 cells and DMEM-12 (Sigma-Aldrich, St. Louis, MO, USA) for PS-1 cells. Media was supplemented with 10% fetal bovine serum (Sigma-Aldrich, St. Louis, MO, USA) and cells were kept at 37 °C and 5% CO_2_ in a humid incubator. To study the effect of agonist and antagonist treatment, AsPC-1 cells were treated with 100 µM of the P2Y_2_ agonist, adenosine 5′-triphosphate disodium salt hydrate (Sigma-Aldrich, St. Louis, MO, USA) and 5 µM of the P2Y_2_ antagonist AR-C 118925XX (Tocris, Bristol, UK) for 1 h before fixation.

### 2.2. Cell Fixation and Immunofluorescence Staining for Antibody Validation

AsPC-1 and PS-1 cells were seeded in coverslips placed in a 6 well-plate (Corning, Corning, NY, USA) at a seeding density of 200,000 cells per well. Cells were fixed in 4% paraformaldehyde (LifeTech, Carlsbad, CA, USA) for 30 min and washed 3× in PBS. Cells were permeabilised with 0.1% Triton X-100 (Avantor, Radnor Township, PA, USA) and incubated for 10 min. The coverslips were washed 3× with PBS. Blocking was performed using 5% bovine serum albumin (Merck, Kenilworth, NJ, USA) in PBS for 1 h. To image P2Y_2_ receptors, the anti-P2Y_2_ receptor antibody (APR-010, Alomone labs, Jerusalem, Israel), which binds to the third intracellular loop between the fifth and sixth transmembrane domain of P2Y_2,_ was used for immunostaining. The P2Y_2_ antibody was diluted in blocking solution at 1:200 as previously reported [32]. Cells were incubated overnight at 4 °C with the antibody dilution followed by 3× PBS washes. Then, the secondary antibody Alexa Fluor 546 goat anti-rabbit (Invitrogen, Waltham, MA, USA) was added (1:1000) and left incubating for 1 h. For nuclear staining, 4′,6-diamidino-2-phenylindole (DAPI, Sigma-Aldrich, St. Louis, MO, USA) dilution (1:1000) was added and incubated for 10 min. Slides were mounted with coverslips using Mowiol (Calbiochem, San Diego, CA, USA). Fluorescence images were taken with a Zeiss LSM 710 confocal microscope (Zeiss, Oberkochen, Germany).

### 2.3. DNA—Antibody Coupling Reaction

DNA labelling of anti-P2Y_2_ receptor antibody (APR-010, Alomone labs, Jerusalem, Israel) was performed via maleimidePEG2-succinimidyl ester coupling reaction [20,33]. Briefly, 13 µL of 1 mM thiolated DNA (5′-Thiol-AAACCACCACCACCA-3′, Eurofins, Ebersberg, Germany) was reduced by mixing it on a shaker with 30 µL of a freshly prepared 250 mM DDT (Thermo Fisher Scientific, Waltham, MA, USA) solution for 2 hrs. 30 min after the reduction of the thiol-DNA started, 175 µL of 0.8 mg/mL primary antibody was incubated with 0.9 µL of 23.5 mM maleimide-PEG2-succinimidyl ester cross-linker solution (Sigma-Aldrich, St. Louis, MO, USA) on a shaker for 90 min at 4 °C in the dark. To remove excess DDT and cross-linker, both reactions were purified by spin filtration using a Microspin Illustra G-25 columns (GE Healthcare, Chicago, IL, USA) and a Zeba spin desalting column (7K MWCO, Thermo Fisher Scientific, Waltham, MA, USA), respectively. The resultant products were mixed and incubated on a shaker overnight at 4 °C in the dark. Finally, excess DNA was removed via Amicon spin filtration (100K, Merck, Kenilworth, NJ, USA) and antibody-DNA concentration was measured using a NanoDrop One spectrophotometer (Thermo Fisher Scientific, Waltham, MA, USA).

### 2.4. Cell Fixation and Immunofluorescence Staining for DNA-PAINT Imaging

AsPC-1 cells were seeded at 50% confluency on six-channel glass bottomed microscopy chambers (µ-SlideVI^0.5^, Ibidi, Fitchburg, WI, USA) and pre-fixed for 30 min with pre-warmed 4% paraformaldehyde (LifeTech, Carlsbad, CA, USA) at room temperature. Following 3× washes in PBS, cell membranes were permeabilised for 5 min in 0.1% Triton X-100 solution (Avantor, Radnor Township, PA, USA) and washed 3× in PBS. Auto-fluorescence was quenched using 50 mM ammonium chloride solution (Avantor, Radnor Township, PA, USA) for 5–10 min and washed 3× in PBS. Cells were then blocked in 5% bovine serum albumin (Merck, Kenilworth, NJ, USA) for 60 min and subsequently incubated with the DNA-labelled anti-P2Y_2_ receptor antibody diluted in blocking buffer overnight at 4 °C. The next day, cells were washed 3× in PBS prior to incubation with 150 nm gold nanoparticles (Sigma-Aldrich, St. Louis, MO, USA) which were used as fiducial markers. A 1 nM imager strand solution was made in the presence of an oxygen scavenging and triplet state quencher system consisting of 1× PCA (Stock 40× PCA solution), 1× PCD (Stock 100× PCD solution), and 1× Trolox (Stock 100× Trolox solution) in 1× PBS + 500 mM NaCl buffer and incubated in the dark for 1 h 40× PCA (protocatechuic acid) stock was made from 154 mg of PCA (Sigma-Aldrich, St. Louis, MO, USA) in 10 mL of Ultrapure Distilled water (Invitrogen, Waltham, MA, USA) adjusted to pH 9.0 with NaOH (Avantor, Radnor Township, PA, USA). 100x PCD (protocatechuate 3,4-dioxygenase) solution was made by adding 2.2 mg of PCD (Sigma-Aldrich, St. Louis, MO, USA) to 3.4 mL of 50% glycerol (Sigma-Aldrich, St. Louis, MO, USA) with 50 mM KCl (Sigma-Aldrich, St. Louis, MO, USA), 1 mM EDTA (Invitrogen, Waltham, MA, USA), and 100 mM Tris buffer (Avantor, Radnor Township, PA, USA). 100x Trolox solution was made by dissolving 100 mg of Trolox (Sigma-Aldrich, St. Louis, MO, USA) in 0.43 mL methanol (Sigma-Aldrich, St. Louis, MO, USA), 0.345 mL 1 M NaOH, and 3.2 mL of Ultrapure Distilled water (Invitrogen, Waltham, MA, USA). After gold nanoparticle incubation, cells were washed again 3× in PBS and immediately imaged using the imager strand solution. The imager DNA (5′-TGGTGGT-3′) strand was conjugated to the fluorescent molecule Atto643 at the 3′ terminus.

### 2.5. DNA-PAINT Imaging Experiments

AsPC-1 cells were imaged on a custom built total internal reflection fluorescence (TIRF) microscope based on a Nikon Eclipse Ti-2 microscope (Nikon Instruments, Tokyo, Japan) equipped with a 100× oil immersion TIRF objective (Apo TIRF, NA 1.49) and a Perfect Focus System. Samples were imaged under flat-top TIRF illumination with a 647 nm laser (Coherent OBIS LX, 120 mW, Santa Clara, CA, USA), magnified with both a custom-built telescope (AC254-050-A-ML and AC508-075-A-ML, Thorlabs, Newton, NJ, USA) and a variable beam expander (BE02-05-A, Thorlabs, Newton, NJ, USA), before passing through a beam shaper device (piShaper 6_6_VIS, AdlOptica, Berlin, Germany) to transform the Gaussian profile of the beam into a collimated flat-top profile. Laser polarization was adjusted to circular using a polarizer (LPVISC050-MP2, Thorlabs, Newton, NJ, USA) followed by a quarter waveplate (LAS-043013, Laser 2000, Cambridge, UK). The beam was focused into the back focal plane of the microscope objective using a suitable lens (AC508-300-A-ML, Thorlabs, Newton, NJ, USA), passed through a clean-up filter (FF01-390/482/563/640-25, Semrock, Rochester, NY, USA) and coupled into the objective using a beam splitter (Di03-R405/488/561/635-t1-25×36, Semrock, Rochester, NY, USA). Fluorescence light was spectrally filtered with an emission filter (FF01-446/523/600/677-25, Semrock, Rochester, NY, USA) and imaged on a sCMOS camera (ORCA-Flash4.0 V3 Digital, Hamamatsu, Hamamatsu City, Japan) without further magnification, resulting in a final pixel size of 130 nm in the focal plane, after 2 × 2 binning. Typically, 15,000 frames were acquired with 100 ms integration time and a laser power density at the sample of 0.5 kW/cm^2^. Schematic representation of the optical set-up is depicted in Figure S1.

### 2.6. DNA-PAINT Image Reconstruction and Cluster Analysis

Images were processed and reconstructed using the Picasso software (Version 0.3.3) [20]. Briefly, single molecule events were identified and localised from the raw fluorescent DNA-PAINT imaging experiments using the ‘Localize’ module of Picasso. Subsequently, images were drift corrected with the ‘Render’ module of Picasso using a stepwise protocol. First, via an image sub-stack cross correlation analysis and then using gold nanoparticles as fiducial markers. Localizations with uncertainties greater than 13 nm were removed while no merging was performed for molecules re-appearing in subsequent frames. Finally, regions of interest (ROIs) of ~4 by 4 µm^2^ were selected within the ‘Render’ module of Picasso and analysed using the implementation of DBSCAN from PALMsiever [34] in MATLAB (2021a) [35]. DBSCAN is a data clustering algorithm that detects clusters of localizations by looking for the minimum number of points (‘minPts’) within a circle with radius epsilon (‘eps’). For ‘eps’, we used the localisation precision of our DNA-PAINT images as determined via the nearest-neighbour based analysis, which was ca. 10 nm for all the images. For ‘minPts’ we chose a parameter in accordance with the binding frequency of the imager strand and the number of recorded frames, in our case this value was set to 10 localisations.

### 2.7. qPAINT Analysis

For qPAINT analysis we used a custom-written MATLAB (2021a) [35] code that analyses the fluorescence time series of each detected localization cluster to estimate the number of P2Y_2_ receptors for each cluster as described previously [21,33]. In short, localizations corresponding to the same cluster were grouped and their time stamps (frame number) used to reconstruct the sequence of dark times per cluster as continuous frame times that did not contain an event. All the dark times per cluster were pooled and used to obtain a normalised cumulative histogram of the dark times which was then fitted with the following exponential function: 1 – exp(t/τ_d_) to estimate the dark time, τ_d_, per cluster. The inverse of the dark time was calculated for each cluster and stored as the qPAINT index of the cluster (*q*_i_).

To estimate the number of P2Y_2_ receptors per cluster, a calibration was performed with the DNA-PAINT data of all cells and verified by analysing DNA-PAINT images of DNA labelled anti-P2Y_2_ receptor antibody deposited on glass slides. For each DNA-PAINT data series a histogram of cluster qPAINT indices for small clusters (i.e., clusters with a maximum point distance of 150 nm) was obtained and fitted with a multi-peak Gaussian that exhibited peaks at multiples of a qPAINT index of 0.012 Hz. This calibration value, corresponding to the qPAINT index for one binding site, *q*_i1_, was used to estimate the number of P2Y_2_ receptors per cluster as the ratio of the qPAINT index of the cluster, *q*_i_ and *q*_i1._ To recover a likely distribution of P2Y_2_ receptors in each cluster of localizations, we used *k*-means clustering, where *k* corresponds to the protein copy number per cluster.

### 2.8. Statistical Analysis

For DNA-PAINT imaging, a minimum of twenty-five ~4 by 4 µm^2^ regions obtained from 7–9 AsPC-1 cells were analysed per condition (control, agonist, antagonist). Statistical analysis was performed via R (Version 4.0.3, The R Foundation, Vienna, Austria) using the rstatix package [36,37]. Distribution of data points and their variance were determined. Groups of three independent conditions were compared using non-parametric pairwise Wilcoxon rank sum tests using the Holm correction method for multiple hypothesis testing. Differences were statistically significant when adjusted *p* < 0.05. (n.s., *p* > 0.05; * *p* ≤ 0.05; ** *p* ≤ 0.01; *** *p* ≤ 0.001). Plots were created in R using the packages ggplot2, ggpubr, tidyverse, and ggprism.

## 3. Results

### 3.1. Super-Resolution Imaging of P2Y_2_ Receptors in AsPC-1 Cells Using DNA-PAINT

**Figure 1:**
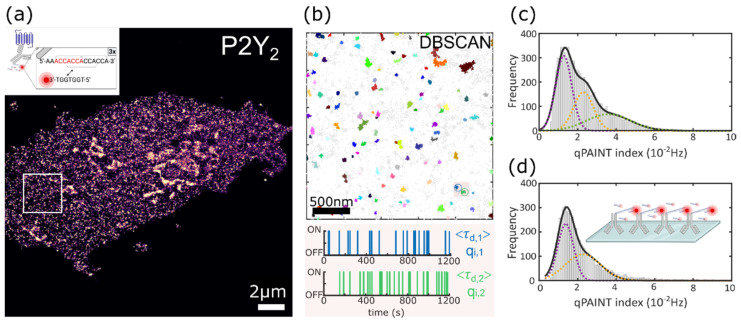
qPAINT calibration to determine *qi*. (**a**) Representative rendered DNA-PAINT image of P2Y_2_ proteins in an AsPC-1 cell. Inset shows a schematic representation of DNA docking and imager strand sequences displaying the 3× repeat binding motif (created with BioRender.com). White box indicates ROI for subsequent cluster analysis. (**b**) DBSCAN cluster analysis output for ROI indicated in (**a**). Non-clustered points are shown in grey. Box represents clusters used for subsequent single molecule on-off time series. (**c**) Histogram of internal calibration qPAINT indexes per cluster pooled from all data samples fit with a multi-Gaussian function. (**d**) Histogram of calibration sample qPAINT indexes per cluster from DNA coupled anti P2Y_2_ antibody deposited on glass fit with a multi-Gaussian function.

To unravel the molecular organization of P2Y_2_ receptors in and near the plasma membrane of AsPC-1 cells, we used DNA-PAINT imaging under total internal reflection (TIR) excitation (see Figure S1 for a schematic representation of the optical set-up). Figure 1a shows a representative super-resolution image of P2Y_2_ obtained via DNA-PAINT. TIR excitation allows investigation of samples at or near the cell membrane by optically sectioning light illumination to only the most superficial ~100 nm of the sample. This is extremely beneficial in the study of GPCRs located at the plasma membrane as the receptors are typically not only at the cell membrane, but also at intracellular sites such as endosomes, endoplasmic reticulum, and the Golgi complex [38] and TIRF imaging minimizes their intracellular visualisation.

P2Y_2_ receptors were labelled with a primary antibody which we validated using the immortalised human pancreatic stellate cell line PS-1 as it expresses traces amounts of the protein of interest (see Figure S2 for negative control results) [19,39]. For DNA-PAINT imaging, the anti-P2Y_2_ receptor antibody was chemically coupled to an optimised docking strand sequence design to increase imaging speed. The docking strand features a repetitive (ACC)n sequence motif that provides 3× overlapping binding sites for the imager strand (Figure 1a, inset) [40]. The benefit of such docking sequence is not only the increase of imaging speed, but also the possibility to use relatively low imager strand concentrations achieving a high signal-to-noise ratio and single-molecule localisation precision. In our experiments we achieved an overall localisation precision of ~10 nm using a 1 nM concentration of imager strand fluorescently labelled with ATTO643, as determined by the nearest-neighbour-based analysis (Figure S3).

### 3.2. Quantitative Analysis of the P2Y_2_ Receptors Oligomerization via qPAINT Analysis

#### 3.2.1. qPAINT Calibration

To investigate the nanoscale distribution and oligomerization state of P2Y_2_ receptors in AsPC-1 cells, we subjected the DNA-PAINT data to qPAINT analysis. qPAINT allows quantification of the exact number of antibody-labelled P2Y_2_ receptors within a cluster of single molecule localisations by taking advantage of the first order binding kinetics between individual imager and docking strands. Specifically, the method uses the average dark time, τ_d_, of a cluster of single molecule localizations to determine how many copies of the protein reside within that cluster of points, *N*, via the following relationship

*N* = (*k*_on_ c_i_ τ_d_)^−1^ = τ_d,1_/τ_d_ (1)

where *k*_on_ is the binding rate of imager to docking strand, c_i_ is the concentration of the imager strand, and τ_d,1_ denotes the dark time for the case of a single protein and is equal to (*k*_on_ c_i_)^−1^. By examination of Equation (1), it is straightforward to see that every additional protein residing in the same cluster of localizations will lead to a proportionally shorter τ_d_, as illustrated in Scheme 1. To calculate the dark times associated to each cluster, we developed an automated data processing pipeline based on DBSCAN (density-based spatial clustering of application with noise) [41]. In DNA-PAINT, a DNA-coupled antibody is localised several times, rendering a cluster of localizations around the true position of the labelled proteins, whereas non-specific binding events are detected as non-clustered localizations. Therefore, we first used the clustering algorithm DBSCAN to automatically identify clusters of localizations in all the data sets, as illustrated in Figure 1b. For each detected cluster, we then calculated the average dark time between binding events by fitting cumulative histograms of the dark time durations, as detailed in the Methods section. Figure 1b presents two typical single molecule on/off time series sequences for the highlighted cluster of localizations containing either one or two docking sites, respectively. To provide a measurement that is directly proportional to the copies of labelled proteins per cluster, *N*, we used the inverse of the measured dark time, a term known as the qPAINT index, *q_i_* [42].

Figure 1c shows a histogram of the qPAINT indexes obtained from the DNA-PAINT data acquired in AsPC-1 cells. This was achieved by selecting very small clusters in the biological data set, based on their geometrical dimension, such that they visually contain one, two, or several puncta. The qPAINT index histogram of P2Y_2_ receptors can be fitted with a multi-Gaussian function with peaks located at multiples of a qPAINT index value of *q_i_*_1_ 0.012 Hz. This ‘quantal’ behaviour is characteristic of detecting one, two, or three units of DNA docking strands in the same cluster of single molecule localisations. Similar results were obtained when examining the qPAINT index histogram of a dedicated calibration sample corresponding to DNA-coupled primary antibodies deposited on a glass coverslip and imaged via DNA-PAINT with the same experimental conditions as the biological experiments (Figure 1d). In this case, the qPAINT index histogram contains the information of all the clusters detected in the calibration sample and exhibits a prominent peak at a small qPAINT index value of *q_i_*_1_ 0.011 Hz and a secondary peak located at nearly double of this value. The consistency between the qPAINT index value for a single docking strand obtained in the calibration sample and the internal calibration of the P2Y_2_ data are a strong indication of the accuracy of the *q_i_*_1_ value 0.012 Hz obtained in the biological samples. This value was thus used to estimate the number of P2Y_2_ receptors in DNA-PAINT images as the ratio between *q*_i_/*q_i_*_1_. This analysis also confirmed that it is possible to differentiate between monomers, dimers, and higher order clusters of antibody-labelled P2Y_2_ receptors using qPAINT analysis.

#### 3.2.2. Comparison of P2Y_2_ Receptor Dimerization and Cluster Formation upon Agonist and Antagonist Treatment of AsPC-1 Cells

**Figure 2:**
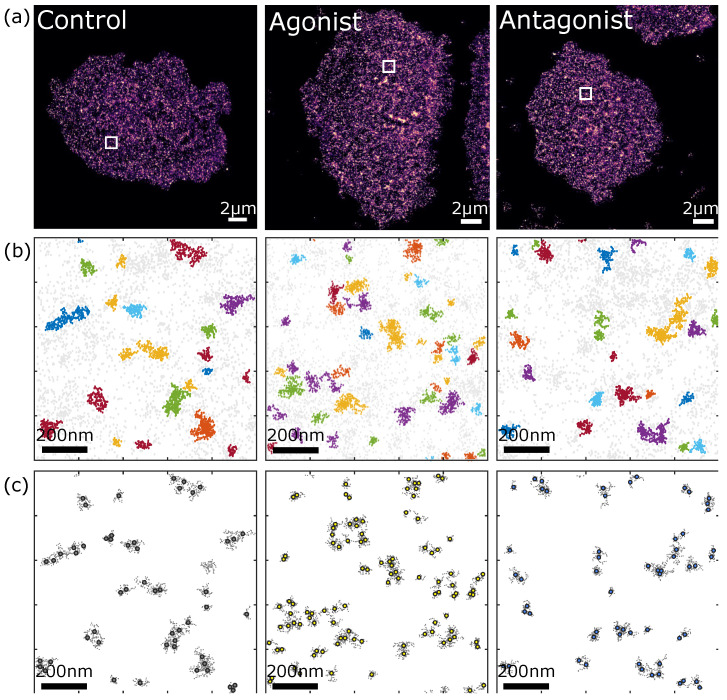
Automated data processing procedure to localize and quantify P2Y_2_ receptor nanoscale distribution. (**a**) Representative rendered DNA-PAINT images of P2Y_2_ proteins in AsPC-1 cells under control, agonist, and antagonist treatments. White box indicates ROI for subsequent cluster analysis displayed in (**b**). (**b**) DBSCAN cluster analysis output for ROIs indicated in (**a**) under control, agonist, and antagonist treatments. Non-clustered points are shown in grey. (**c**) Quantitative protein maps of the ROIs indicated in (**a**,**b**) displaying P2Y_2_ receptors (larger coloured points) in each cluster overlayed on clustered DNA-PAINT single-molecule localizations. (Left to right) control conditions indicated in grey, agonist conditions in yellow and antagonist conditions in blue.

Next, we aimed to demonstrate the applicability of qPAINT analysis to quantitatively determine the agonist and antagonist dependent oligomerization state of the P2Y_2_ receptor in single AsPC-1 cells. Figure 2 illustrates our automated data processing procedure. First, we acquired DNA-PAINT images of the P2Y_2_ receptor in control AsPC-1 cells and cells treated with agonist (ATP) and antagonist (AR-C 118925XX). Examples of these super-resolution images are presented in Figure 2a. To statistically quantify the spatial organisation of the GPCR in different conditions, we used DBSCAN to detect clusters of single-molecule localisations in at least twenty five ~4 by 4 µm^2^ randomly selected regions of interest (ROIs) obtained from 7–9 cells per condition (i.e., control, agonist, and antagonist treated cells). After identifying clusters of localisations in each ROI (colour coded in Figure 2b), we determined the dark time associated with each cluster and estimated the copy number of P2Y_2_ receptors per cluster using the calibration value obtained as described in Section 3.2.1. We used this information to quantify the density of antibody labelled receptors, the percentage of receptors organised into clusters of proteins (clusters defined as an assembly containing more than 5 copies of the receptor), the percentage of receptors forming dimers, and the percentage of non-clustered P2Y_2_ receptors, defined as monomers. Furthermore, we then used *k*-means clustering (a distance-based algorithm) to partition the clusters of single molecule localisations into *k* clusters, where *k* is given by the copy number of receptors per cluster. This pipeline, previously introduced by Simoncelli et al. [33], allowed us to recover an accurate quantitative map of the nanoscale distribution of labelled P2Y_2_ receptors in AsPC-1 cells (Figure 2c). Using *k*-means clustering, we were also able to calculate that the mean distance between two P2Y_2_ receptors in the clusters identified as dimers is 25 ± 10 nm (Figure S4). Considering both the physical size of the labelling antibodies (13.7 nm) [43] and the localization error (10 nm, Figure S3), the furthest apart two adjacent antibodies could be is ca. 27 nm ± 10 nm, which is within the range of the measured dimer distance, suggesting that a distance of 25 ± 10 nm is likely to correspond to the detection of true chemical dimerization.

**Figure 3:**
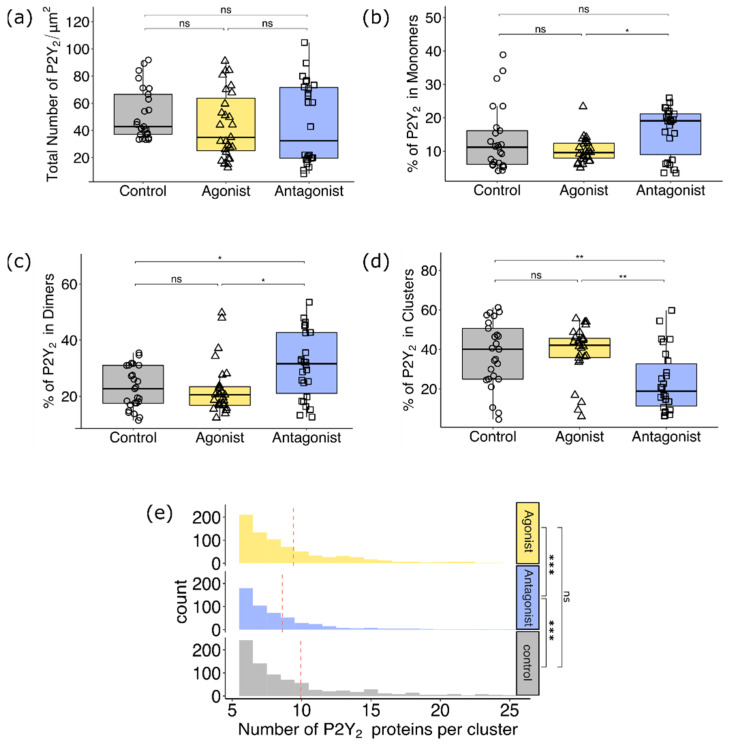
Comparison of P2Y_2_ protein clustering in AsPC-1 cells under control, agonistic and antagonistic conditions. (**a**) Density of P2Y_2_ proteins under control, agonistic and antagonistic conditions. (**b**) Percentage of P2Y_2_ detected as monomers under control, agonistic and antagonistic conditions. (**c**) Percentage of P2Y_2_ forming dimers under control, agonistic and antagonistic conditions. (**d**) Percentage of P2Y_2_ receptors forming clusters of more than 5 proteins under control, agonistic and antagonistic conditions. (**e**) Number of P2Y_2_ proteins per cluster under control, agonistic, and antagonistic conditions. Control conditions depicted in grey with circular points, agonist in yellow with triangular points and antagonist in blue with square points. Significance values from paired Wilcoxon sum tests adjusted *p* values >0.05 = ns, <0.05 = *, <0.01 = **, <0.001 = ***. *n* = 25, 27, and 26 ROIs, respectively.

Using this approach, we found that the density of labelled P2Y_2_ receptors in the plasma membrane (and/or the membrane-proximal area) does not change upon agonist or antagonist treatment (Figure 3a). Quantification of the extent of dimerization and clustering of P2Y_2_ receptors also revealed that the percentage of receptors in monomers, dimers, and clusters are not significantly different between agonist treated and control conditions (Figure 3b–d). Similar trends were observed by Kotevic et al. [29] using FRET measurements of HEK 293 cells co-expressing P2Y_2_-CFP and -YFP, where no significant change in the FRET signal was observed upon exposure to the agonist, in their case UTP. Unlike the comparison between control and agonist treated cells, there is a marked decreased (ca. 50%) in the percentage of P2Y_2_ receptors forming clusters in antagonist treated cells with respect to the control conditions (Figure 3d). The mean P2Y_2_ receptor copy number per cluster was also highly significantly different between control and antagonist and agonist and antagonist treated cells but did not change significantly between agonist and control conditions (Figure 3e). Concurrent with the reduction of the percentage of molecules forming clusters and number of molecules in clusters in antagonist treated cells, this analysis also detected an increase in the percentage of P2Y_2_ receptors forming dimers and in the monomeric state (Figure 3b,c). Together these data indicate that the antagonist prevents the formation of higher order clusters and reduces their size.

## 4. Discussion

Over the years, multiple optical microscopy techniques have been applied to the study of GPCR oligomerization, with one of the first single molecule imaging studies done by Kasai et al. [44]. Subsequently, both Spatial Intensity Distribution Analysis (SpIDA) [15,45] and molecular brightness approaches [13,14] were also developed and applied to study a variety of GPCRs. In recent years, single-molecule tracking and FRET imaging have also been applied to identify key factors in the regulation of GPCRs dynamic interactions in living cells [16,46]. While these techniques have been paramount to investigate the oligomeric organisation of GPCRs and the dynamic interactions that control GPCRs signalling, many challenges still remain; including the limitation of applicability to endogenous settings (i.e., many of these methods require labelled receptors or fusion proteins) and the possibility of multi-colour imaging to simultaneously study different types of hetero-oligomers. Most recently, dSTORM (direct stochastic optical reconstruction microscopy), a single-molecule super-resolution microscopy method, was employed to investigate the nanoscale organisation of a GPCR in presynaptic active zones [47], presenting a step-forward in visualising the organization of these receptors in endogenous conditions with nanoscale resolution. However, this was possible at the cost of increased experimental complexity, as the approach required transiently transfecting cells under controlled conditions to accurately quantify the number of receptors per cluster of single-molecule localizations.

As a novel method for quantitative analysis and nano-scale visualisation of the oligomeric state of GPCRs in single cells, here we employed a quantitative single molecule based super-resolution imaging technique based on DNA-PAINT, named qPAINT. Our approach allows the study of GPCR oligomerization in an endogenous setting, while avoiding the difficulties experienced with other SMLM techniques, such as dSTORM or PALM, to quantify the protein copy number [47,48]. During the last four years, examples of the use of qPAINT to quantify protein clustering in different biological systems have started to emerge [33,42,49]. This work is the first to employ qPAINT towards the study of GPCRs, a technique that can certainly help resolve contentious issues associated with GPCR oligomerization in different settings. Furthermore, due to the comparative advantages of DNA-PAINT over other SMLM techniques—it can routinely deliver lateral resolution in the 5–10 nm range and it allows multiplexing with a single laser source—it is straightforward to expand the applicability of qPAINT to detect and quantify GPCR hetero-oligomers. We note, however, that like any other technique that relies on antibody labelling, its applicability will depend on the availability of validated antibodies and that the final accuracy towards the absolute protein copy number is affected by the overall antibody-target binding efficiency, which can lead to potential undercounting. Still, by keeping data acquisition and analysis the same it is possible to draw conclusions about changes in the nanoscale distribution of the receptors in the different conditions. Furthermore, the presented approach is not suitable for live-cell imaging.

By leveraging the capabilities of qPAINT to its maximum potential, our study provides a detailed quantitative characterization of the density, spatial organization, and stoichiometry of the antibody labelled purinergic receptor Y2 oligomers in the pancreatic cancer cell line AsPC-1. Specifically, our data indicate that P2Y_2_ receptors are highly expressed in AsPC-1 cells with an average density of ca. 40 receptors per µm^2^. We find that P2Y_2_ receptors are mainly organised in nanodomains containing three or more sub-units, with only 20% of the receptors forming dimers and 10% distributed as monomers. Our results also show that while the oligomerization status of P2Y_2_ receptors does not change upon agonist treatment, there is a marked reduction of the percentage of P2Y_2_ receptors forming oligomers in antagonistic conditions. Our results are in line with current models for the P2Y_2_ receptor, which imply that homo-oligomeric assemblies of P2Y_2_ receptors are required for receptor internalisation and the effect of the antagonist prevents the formation of these complexes. Similar antagonist effects have been observed for CXCR4 and dopamine D3 GPCR receptors [50,51]. The fact that we did not observe significant difference in the oligomerisation of P2Y_2_ receptors in control and agonistic conditions suggest that oligomeric assemblies could be part and parcel of the natural activation of the receptor.

In conclusion, we demonstrated the accuracy and ease of implementation of qPAINT to quantitatively characterise the oligomeric state of GPCRs, alongside achieving single receptor visualisation. qPAINT analysis of the oligomeric state of P2Y_2_ revealed consistency with currently proposed models for this receptor [29,50,51].

## Data Availability

The data supporting this research are available upon request.

## Supplementary Materials

The following are available online at www.mdpi.com/article/10.3390/biom11101503/s1, Figure S1: Schematic of custom-built super-resolution microscopy setup. Figure S2: P2Y_2_ and DAPI immunofluorescent staining of AsPC-1 and PS-1 cells for anti-P2Y_2_ receptor antibody validation. Figure S3: Overall localization precision of all super-resolution DNA-PAINT images. Figure S4: Distance between P2Y_2_ receptors in dimers.

## References

[1] Stevens R.C., Cherezov V., Katritch V., Abagyan R., Kuhn P., Rosen H., Wüthrich K. (2013). The GPCR Network: A large-scale collaboration to determine human GPCR structure and function. Nat. Rev. Drug Discov..

[2] Israeli H., Degtjarik O., Fierro F., Chunilal V., Gill A.K., Roth N.J., Botta J., Prabahar V., Peleg Y., Chan L.F. (2021). Structure reveals the activation mechanism of the MC4 receptor to initiate satiation signaling. Science.

[3] Jagannath A., Varga N., Dallmann R., Rando G., Gosselin P., Ebrahimjee F., Taylor L., Mosneagu D., Stefaniak J., Walsh S. (2021). Adenosine integrates light and sleep signalling for the regulation of circadian timing in mice. Nat. Commun..

[4] Sriram K., Insel P.A. (2018). G protein-coupled receptors as targets for approved drugs: How many targets and how many drugs?. Mol. Pharmacol..

[5] Hill R., Canals M. (2021). Experimental considerations for the assessment of in vivo and in vitro opioid pharmacology. Pharmacol. Ther..

[6] Gillis A., Gondin A.B., Kliewer A., Sanchez J., Lim H.D., Alamein C., Manandhar P., Santiago M., Fritzwanker S., Schmiedel F. (2020). Low intrinsic efficacy for G protein activation can explain the improved side effect profiles of new opioid agonists. Sci. Signal..

[7] Pandey S., Kumari P., Baidya M., Kise R., Cao Y., Dwivedi-Agnihotri H., Banerjee R., Li X.X., Cui C.S., Lee J.D. (2021). Intrinsic bias at non-canonical, beta-arrestin-coupled seven transmembrane receptors. Mol. Cell.

[8] Stahl E.L., Bohn L.M. (2021). Low Intrinsic Efficacy Alone Cannot Explain the Improved Side Effect Profiles of New Opioid Agonists. Biochemistry.

[9] Guo X., Li Q., Pi S., Xia Y., Mao L. (2021). G protein-coupled purinergic P2Y receptor oligomerization: Pharmacological changes and dynamic regulation. Biochem. Pharmacol..

[10] Bouvier M. (2001). Oligomerization of G-protein-coupled transmitter receptors. Nat. Rev. Neurosci..

[11] Guo H., An S., Ward R., Yang Y., Liu Y., Guo X.X., Hao Q., Xu T.R. (2017). Methods used to study the oligomeric structure of G-protein-coupled receptors. Biosci. Rep..

[12] El Khamlichi C., Reverchon-Assadi F., Hervouet-Coste N., Blot L., Reiter E., Morisset-Lopez S. (2019). Bioluminescence resonance energy transfer as a method to study protein-protein interactions: Application to G protein coupled receptor biology. Molecules.

[13] Işbilir A., Serfling R., Möller J., Thomas R., De Faveri C., Zabel U., Scarselli M., Beck-Sickinger A.G., Bock A., Coin I. (2021). Determination of G-protein–coupled receptor oligomerization by molecular brightness analyses in single cells. Nat. Protoc..

[14] Stoneman M.R., Biener G., Ward R.J., Pediani J.D., Badu D., Eis A., Popa I., Milligan G., Raicu V. (2019). A general method to quantify ligand-driven oligomerization from fluorescence-based images. Nat. Methods.

[15] Godin A.G., Costantino S., Lorenzo L.E., Swift J.L., Sergeev M., Ribeiro-da-Silva A., De Koninck Y., Wiseman P.W. (2011). Revealing protein oligomerization and densities in situ using spatial intensity distribution analysis. Proc. Natl. Acad. Sci. USA.

[16] Sungkaworn T., Jobin M.L., Burnecki K., Weron A., Lohse M.J., Calebiro D. (2017). Single-molecule imaging reveals receptor-G protein interactions at cell surface hot spots. Nature.

[17] Calebiro D., Rieken F., Wagner J., Sungkaworn T., Zabel U., Borzi A., Cocucci E., Zürn A., Lohse M.J. (2013). Single-molecule analysis of fluorescently labeled G-protein-coupled receptors reveals complexes with distinct dynamics and organization. Proc. Natl. Acad. Sci. USA.

[18] Scarselli M., Annibale P., McCormick P.J., Kolachalam S., Aringhieri S., Radenovic A., Corsini G.U., Maggio R. (2015). Revealing G-protein-coupled receptor oligomerization at the single-molecule level through a nanoscopic lens: Methods, dynamics and biological function. FEBS J..

[19] Hu L.-P., Zhang X.-X., Jiang S.-H., Tao L.-Y., Li Q., Zhu L.-L., Yang M.-W., Huo Y.-M., Jiang Y.-S., Tian G.-A. (2019). Targeting Purinergic Receptor P2Y2 prevents the growth of pancreatic ductal adenocarcinoma by inhibiting cancer cell glycolysis. Clin. Cancer Res..

[20] Schnitzbauer J., Strauss M.T., Schlichthaerle T., Schueder F., Jungmann R. (2017). Super-resolution microscopy with DNA-PAINT. Nat. Protoc..

[21] Jungmann R., Avendaño M.S., Dai M., Woehrstein J.B., Agasti S.S., Feiger Z., Rodal A., Yin P. (2016). Quantitative super-resolution imaging with qPAINT. Nat. Methods.

[22] Orriss I.R., Guneri D., Hajjawi M.O.R., Shaw K., Patel J.J., Arnett T.R. (2017). Activation of the P2Y2 receptor regulates bone cell function by enhancing ATP release. J. Endocrinol..

[23] Séror C., Melki M.T., Subra F., Raza S.Q., Bras M., Saïdi H., Nardacci R., Voisin L., Paoletti A., Law F. (2011). Extracellular ATP acts on P2Y2 purinergic receptors to facilitate HIV-1 infection. J. Exp. Med..

[24] de la Rosa G., Gómez A.I., Baños M.C., Pelegrín P. (2020). Signaling through purinergic receptor p2y2 enhances macrophage il-1β production. Int. J. Mol. Sci..

[25] Li W., Qiu Y., Zhang H., Tian X., Fang W. (2015). P2Y2 Receptor and EGFR Cooperate to Promote Prostate Cancer Cell Invasion via. PLoS ONE.

[26] Zhang J., Liu Y., Yang H., Zhang H., Tian X. (2017). ATP-P2Y2- b -catenin axis promotes cell invasion in breast cancer cells. Cancer Sci..

[27] Woods L.T., Jasmer K.J., Muñoz Forti K., Shanbhag V.C., Camden J.M., Erb L., Petris M.J., Weisman G.A. (2020). P2Y2 receptors mediate nucleotide-induced EGFR phosphorylation and stimulate proliferation and tumorigenesis of head and neck squamous cell carcinoma cell lines. Oral Oncol..

[28] Abe M., Watanabe K., Kuroda Y., Nakagawa T., Higashi H. (2018). Homodimer formation by the ATP/UTP receptor P2Y2 via disulfide bridges. J. Biochem..

[29] Kotevic I., Kirschner K.M., Porzig H., Baltensperger K. (2005). Constitutive interaction of the P2Y2 receptor with the hematopoietic cell-specific G protein Gα16 and evidence for receptor oligomers. Cell. Signal..

[30] Tan M.H., Shimano T., Chu T.M. (1981). Differential localization of human pancreas cancer-associated antigen and carcinoembryonic antigen in homologous pancreatic tumoral xenograft. J. Natl. Cancer Inst..

[31] Froeling F.E.M., Mirza T.A., Feakins R.M., Seedhar A., Elia G., Hart I.R., Kocher H.M. (2009). Organotypic culture model of pancreatic cancer demonstrates that stromal cells modulate E-cadherin, β-catenin, and ezrin expression in tumor cells. Am. J. Pathol..

[32] Choi R.C.Y., Chu G.K.Y., Siow N.L., Yung A.W.Y., Yung L.Y., Lee P.S.C., Lo C.C.W., Simon J., Dong T.T.X., Barnard E.A. (2013). Activation of utp-sensitive p2y2 receptor induces the expression of cholinergic genes in cultured cortical neurons: A signaling cascade triggered by ca2+ mobilization and extracellular regulated kinase phosphorylations. Mol. Pharmacol..

[33] Simoncelli S., Griffié J., Williamson D.J., Bibby J., Bray C., Zamoyska R., Cope A.P., Owen D.M. (2020). Multi-color Molecular Visualization of Signaling Proteins Reveals How C-Terminal Src Kinase Nanoclusters Regulate T Cell Receptor Activation. Cell Rep..

[34] Pengo T., Holden S.J., Manley S. (2015). PALMsiever: A tool to turn raw data into results for single-molecule localization microscopy. Bioinformatics.

[35] MATLAB (2021). Version 9.9.0 (R2021a).

[36] R Core Team (2019). R: A Language and Environment for Statistical Computing.

[37] Allaire J. (2011). RStudio: Integrated Development Environment for R.

[38] Sposini S., Hanyaloglu A.C. (2017). Spatial encryption of G protein-coupled receptor signaling in endosomes; Mechanisms and applications. Biochem. Pharmacol..

[39] Sriram K., Salmerón C., Wiley S.Z., Insel P.A. (2020). GPCRs in pancreatic adenocarcinoma: Contributors to tumour biology and novel therapeutic targets. Br. J. Pharmacol..

[40] Strauss S., Jungmann R. (2020). Up to 100-fold speed-up and multiplexing in optimized DNA-PAINT. Nat. Methods.

[41] Ester M., Kriegel H.-P., Sander J., Xu X. A Density-Based Algorithm for Discovering Clusters in Large Spatial Databases with Noise. Proceedings of the 2nd International Conference on Knowledge Discovery and Data Mining.

[42] Jayasinghe I., Clowsley A.H., Lin R., Lutz T., Harrison C., Green E., Baddeley D., Di Michele L., Soeller C. (2018). True Molecular Scale Visualization of Variable Clustering Properties of Ryanodine Receptors. Cell Rep..

[43] Tan Y.H., Liu M., Nolting B., Go J.G., Gervay-Hague J., Liu G.Y. (2008). A nanoengineering approach for investigation and regulation of protein immobilization. ACS Nano.

[44] Kasai R.S., Suzuki K.G.N., Prossnitz E.R., Koyama-Honda I., Nakada C., Fujiwara T.K., Kusumi A. (2011). Full characterization of GPCR monomer-dimer dynamic equilibrium by single molecule imaging. J. Cell Biol..

[45] Ward R.J., Pediani J.D., Godin A.G., Milligan G. (2015). Regulation of oligomeric organization of the serotonin 5-hydroxytryptamine 2C (5-HT2C) receptor observed by spatial intensity distribution analysis. J. Biol. Chem..

[46] Asher W.B., Geggier P., Holsey M.D., Gilmore G.T., Pati A.K., Meszaros J., Terry D.S., Mathiasen S., Kaliszewski M.J., McCauley M.D. (2021). Single-molecule FRET imaging of GPCR dimers in living cells. Nat. Methods.

[47] Siddig S., Aufmkolk S., Doose S., Jobin M.L., Werner C., Sauer M., Calebiro D. (2020). Super-resolution imaging reveals the nanoscale organization of metabotropic glutamate receptors at presynaptic active zones. Sci. Adv..

[48] Jonas K.C., Fanelli F., Huhtaniemi I.T., Hanyaloglu A.C. (2015). Single molecule analysis of functionally asymmetric G protein-coupled receptor (GPCR) oligomers reveals diverse spatial and structural assemblies. J. Biol. Chem..

[49] Fischer L.S., Klingner C., Schlichthaerle T., Strauss M.T., Böttcher R., Fässler R., Jungmann R., Grashoff C. (2021). Quantitative single-protein imaging reveals molecular complex formation of integrin, talin, and kindlin during cell adhesion. Nat. Commun..

[50] Marsango S., Caltabiano G., Jiménez-Rosés M., Millan M.J., Pediani J.D., Ward R.J., Milligan G. (2017). A molecular basis for selective antagonist destabilization of dopamine D3 receptor quaternary organization. Sci. Rep..

[51] Ward R.J., Pediani J.D., Marsango S., Jolly R., Stoneman M.R., Biener G., Handel T.M., Raicu V., Milligan G. (2021). Chemokine receptor CXCR4 oligomerization is disrupted selectively by the antagonist ligand IT1t. J. Biol. Chem..

