# Supplementary material for: Quantitative Super-Resolution Imaging for the Analysis of GPCR Oligomerization
Source: Biomolecules. 2021 Oct 12;11(10):1503. doi: 10.3390/biom11101503 (PMC8533726; doi:10.3390/biom11101503)
Supplement: Supplementary file 1 [file biomolecules-11-01503-s001.zip › biomolecules-1386753-supplementary.pdf]

Supplementary Material

Quantitative super-resolution imaging for the analysis of GPCR oligomerization.

Megan D. Joseph, Elena Tomas Bort, Richard P. Grose, Peter J. McCormick and Sabrina Simoncelli

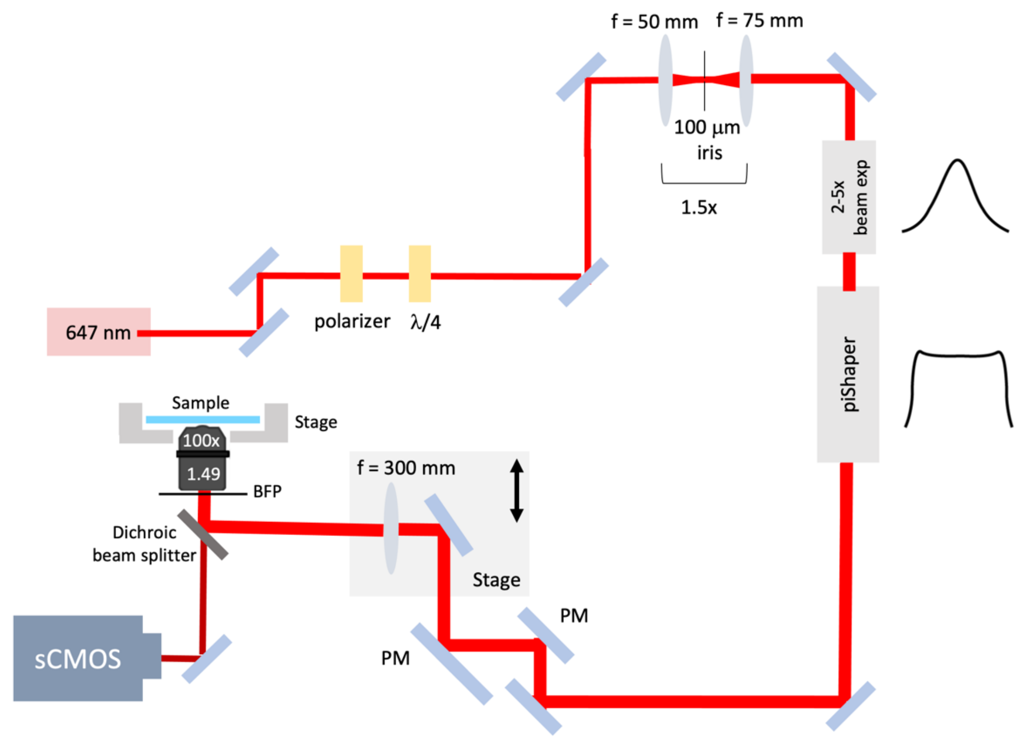

Figure S1. Schematic of custom-built super-resolution microscopy setup. Blue rectangles indicate dielectric mirrors, ellipses indicate lenses.

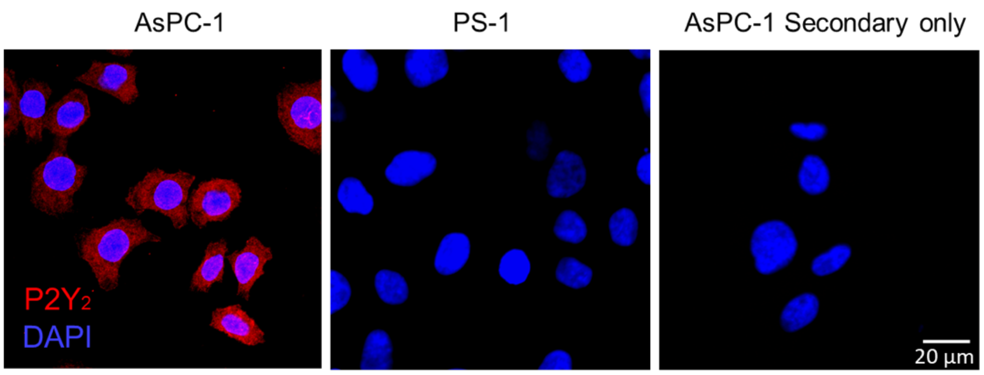

Figure S2. $P2Y_2$ and DAPI immunofluorescent staining of AsPC-1 and PS-1 cells for anti-$P2Y_2$ receptor antibody validation. AsPC-1 cells show bright red $P2Y_2$ staining compared to the low $P2Y_2$ expressing PS-1 cell line and a control using secondary antibody staining only. Images were taken with a confocal microscope. Contrasts setting are the same for all images.

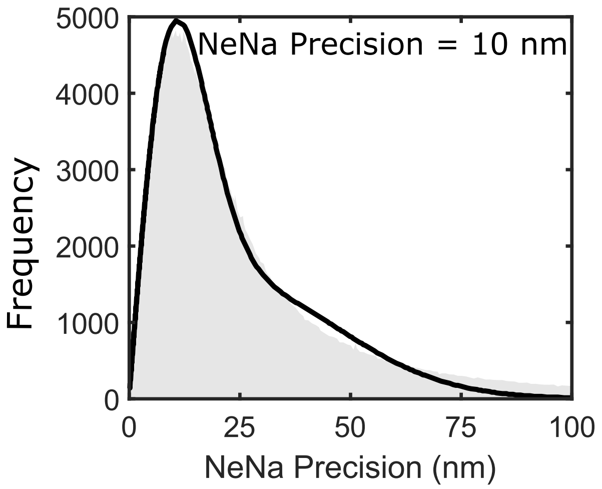

Figure S3. Overall localization precision of all super-resolution DNA-PAINT images. Nearest-Neighbour (NeNa) based analysis was used to determine this precision number.

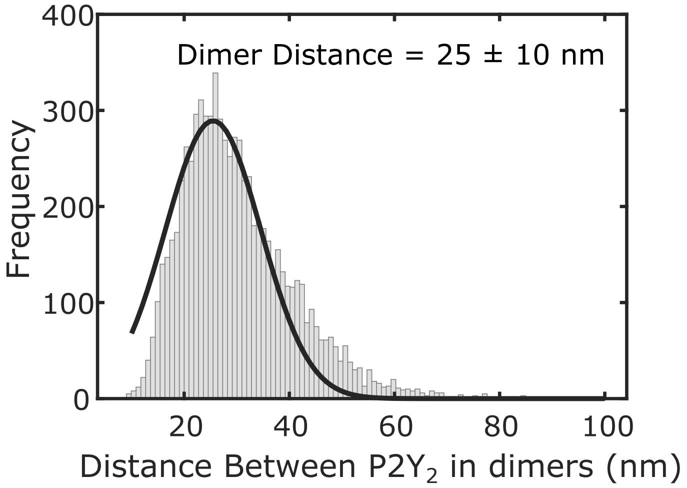

Figure S4. Distance between $P2Y_2$ receptors in dimers. Calculated from $P2Y_2$ receptors in dimers - pooled from all ROIs in every condition (control, agonist and antagonist treated AsPC-1 cells) as determined by the qPAINT analysis pipeline via *k*-means clustering analysis.
